# Validation and Prediction of the School Psychological Capital Among Chinese College Students

**DOI:** 10.3389/fpsyg.2021.697703

**Published:** 2021-07-09

**Authors:** Xia Kang, Yajun Wu, Lisheng Li

**Affiliations:** ^1^Teacher Education and Learning Leadership Unit, Faculty of Education, The University of Hong Kong, Hong Kong, China; ^2^School of Foreign Languages and Literature, Yunnan Normal University, Kunming, China

**Keywords:** school psychological capital, validation, academic engagement, achievement emotions, college students

## Abstract

This study validated the school psychological capital (PsyCap) scale in the Chinese context and examined the predictive effect of PsyCap resources on academic engagement and achievement emotions. Self-report data for PsyCap resources, student engagement, enjoyment, anxiety, and boredom toward English learning were collected from 1,000 sophomores. Item-level analyses and confirmatory factor analysis were used to verify the validity of the school PsyCap scale, and structural equation modeling was applied to reveal the predictive effect of school PsyCap resources on academic engagement and achievement emotions. Results showed that the school PsyCap scale retained superior psychometric properties. Besides, PsyCap resources were demonstrated to have a positive relationship to academic engagement and enjoyment, and a negative relationship to anxiety and boredom. The effectiveness of the school PsyCap scale was verified among Chinese college students, and besides the traditional predictors, school PsyCap is also critically important for students’ academic engagement and achievement emotions. Limitations and implications are discussed.

## Introduction

With the rise of positive psychology in educational research, the school PsyCap with positive psychology as the core has attracted more and more attention from scholars. The notion of PsyCap was firstly proposed in the industrial-organizational contexts to act as the crucial success factor for improving competitiveness and work efficiency (Luthans et al., 2004). Recently, the beneficial impact of PsyCap on academic outcomes (e.g., engagement, achievement emotions, subjective wellbeing, and academic performance) has also been valued in educational settings (Li et al., 2014; Siu et al., 2014; Datu et al., 2018; Carmona-Halty et al., 2019b; King et al., 2020). Psychological captial refers to the positive psychological state or psychological resources displayed in the process of individual growth and development, which is composed of four components of hope, optimism, resilience, and self-efficacy (Luthans et al., 2007b).

Although PsyCap has a significant positive effect on academic outcomes, notably, past studies mainly conducted in western contexts (Carmona-Halty et al., 2019a,b; Martínez et al., 2019), except for Li et al. (2014) study, few studies have focused on the PsyCap of Mainland Chinese students. Besides, prior studies mainly treated PsyCap construct as an observed variable without mitigating the potential measurement errors (Li et al., 2014; Datu and Valdez, 2016). That is, attention should be paid to the synergies among the four subscales of PsyCap rather than merely treating them as independent variables. Under the circumstance that educators are increasingly aware of the importance of building positive psychological resources for students, Chinese students’ PsyCap and its positive effects on achievement emotions and academic engagement need to be further explored.

Taking Chinese university students as participants, the present study aimed to fill these gaps by verifying the validity of the PsyCap scale and the potential synergies among the four subscales in Chinese university context, and exploring whether PsyCap has a predictive effect on the achievement emotions and academic engagement.

## Literature Review

### School Psychological Capital

Psychological captial concentrates on an individual’s positive psychological capacities and advantages. Since the notion of PsyCap was first introduced into the field of education, the characteristics of students’ PsyCap and its relationship with academic outcomes have received educators and practitioners’ extensive attention. However, as PsyCap is a formative measurement model constructed base on the synchronicity of the academic community, its essential characteristics, components, and synergies among components are all controversial.

There are three views on the essential characteristics of PsyCap, namely, trait-like, state-like, and bundling. Hosen et al. (2003) and Peterson and Seligman (2004) regarded PsyCap as a stable internal psychological structure produced by individual self-investment. By considering the developability of PsyCap, Luthans (2002) and Luthans et al. (2007b) argued that PsyCap is a state-like attitude rather than psychological traits. The bundling view of PsyCap holds that PsyCap has both state-like and trait-like aspects (Luthans et al., 2005). Given that all the four components of PsyCap (i.e., self-efficacy, hope, optimism, and resilience) are state-like positive psychological forces, and PsyCap can be cultivated *via* training, PsyCap was usually regarded as malleable and state-like (Luthans et al., 2005, 2007a).

Self-efficacy (or confidence), hope, optimism, and resilience are the four psychological capacities that contribute to the formation of positive PsyCap. Self-efficacy refers to an individual’s conviction that he or she has the ability to execute challenging tasks. As a key psychological resource, self-efficacy can effectively alleviate the negative impact of stress on individuals (e.g., Luthans, 2007b) and has a positive predictive effect on academic performance (Galla et al., 2014; Jiang et al., 2014), optimistic achievement emotions (Putwain et al., 2018), and student motivation (Bong and Clark, 1999; Jiang et al., 2014). Snyder (2000, p. 9) proposed that hope is goal-related thinking, which consists of three components, namely, “goals, pathways, and agency”. The positive predictive effects of hope have been verified in existing studies. For example, Snyder et al. (2002) and Day et al. (2010) took college students as participants and found that hope had a positive association with academic success. Moreover, the contributions of hope to positive emotions (Aspinwall and Leaf, 2002; Khodarahimi, 2013) and psychological adjustment (Du and King, 2013) were also confirmed. From the perspective of attribution theory, Seligman (2002) regarded optimism as the explanatory style for handling events: permanence and pervasiveness. Specifically, the psychological capacity of optimism refers to individuals’ expectation of positive outcomes for their endeavors. The predictive effects of optimism were also vastly explored in the academic context. For instance, optimism has been positively linked to academic performance (Hoy et al., 2006; Feldman and Kubota, 2015; Icekson et al., 2020) and academic engagement (Medlin and Faulk, 2011). Resilience refers to the capacity of sustaining and bouncing back when encountering problems and adversities (Luthans et al., 2007a). That is, students high in resilience are more likely to view adversities as challenges rather than threats (Symes et al., 2015). The positive effects of resilience on academic outcomes have also been widely explored. For example, studies have shown that academic resilience was the positive predictor of classroom participation, enjoyment of school, self-esteem (Martin and Marsh, 2006), academic buoyancy (Martin and Marsh, 2009), academic performance (Kotzé and Kleynhans, 2013; Ayala and Manzano, 2018), and psychological health (García-Izquierdo et al., 2018). To sum up, each component of PsyCap has important and positive significance for students’ academic outcomes.

### Measurement of School Psychological Capital

Diachronically, both the constructs of PsyCap and the interaction mode of these constructs were in the process of dynamic development. Until recent years, there has been a consensus on the constructs of PsyCap within the academic community; however, the higher-order PsyCap instrument in the academic context remains to be explored.

In the early days, the self-esteem scale was used to measure a Person’s PsyCap, that is, the only construct of PsyCap was self-esteem (Goldsmith et al., 1997). Afterward, Letcher and Niehoff (2004) equalized PsyCap to five constructs, those are neuroticism, extraversion, openness, agreeableness, and conscientiousness. Later, Avey et al. (2006) and Larson and Luthans (2006) used four constructs (i.e., self-efficacy, hope, resilience, and optimism) to represent the PsyCap measure. Specifically, the four PsyCap components are composed of Scheier and Carver’s (1985) optimism scale, Wagnild and Young’s (1993) resilience scale, Snyder et al.’s (1996) hope scale, and Parker’s (1998) self-efficacy scale. So far, the use of the four psychological capacities of self-efficacy, hope, resilience, and optimism to characterize PsyCap has become a consensus of the academic circles (Luthans et al., 2007b), and its application scope has also begun to expand from the industrial-organizational contexts to the school contexts.

Except for King and Caleon’s (2021) study, very few studies have explored the psychometric properties of the PsyCap scale in school contexts. King and Caleon (2021) firstly adapted the items in Luthans et al.’s (2007a) PsyCap scale into school-related ones, and then tested the validity of the school PsyCap scale with Singapore secondary students as participants. The overall Cronbach’s alpha reliability of the school PsyCap scale was *α* = 0.91, and the model fit of the scale was adequate. However, the school PsyCap scale developed by King and Caleon (2021) was only verified in the Singapore context and this questionnaire was presented in English, less is known about the validity of this scale in non-English-speaking countries, especially China.

According to the constitutional forms of the items, four competing models of school PsyCap scale coexist, namely, the null model, the unidimensional model, the four-factor model, and the hierarchical model. For example, Li et al. (2014) obtained the value of PsyCap by summing-up and averaging the values of the items, PsyCap was regarded as an omnibus construct, but the measurement error was ignored. Datu and Valdez (2016) and Carmona-Halty et al. (2019b) posited the school PsyCap scale as a unidimensional structure, that is, the four components of the PsyCap scale were treated as observational variables. Recently, Datu et al. (2018) and King et al. (2020) viewed school PsyCap structure as a hierarchical model, that is, both PsyCap and its four components were posited as latent constructs. In the Singapore context, King and Caleon (2021) conducted confirmatory factor analyses to examine the fit of the unidimensional model, the four-factor model, and the hierarchical model of the school PsyCap scale and found that the hierarchical model was the most acceptable one. Given the coexistence of multiple models of school PsyCap scale, more research is needed to examine which model has the best fit.

### The Present Study

The present study had two objectives. The first one was to examine the construct validity of the school PsyCap scale among the Chinese population. Specifically, the psychometric properties and factor structure of the school PsyCap scale were tested with Chinese college students as participants. The second objective was to examine whether school PsyCap was the predictor of the outcome variables of academic engagement and achievement emotions among Chinese college students.

Both in industrial-organizational contexts and school contexts, the previous studies argued that the PsyCap scale has the best fitting degree when it was regarded as a hierarchical model (Luthans et al., 2007a; Datu et al., 2018; King and Caleon, 2021). The hierarchical model posits PsyCap as a second-order latent variable with self-efficacy, hope, resilience, and optimism as its first-order latent factors. Accordingly, we formed the first hypothesis of this study.

*H1*: The hierarchical model of the school PsyCap scale would have the best model fit in the Chinese university context. More clearly, this study posited that school PsyCap would be a second-order latent factor with self-efficacy, resilience, hope, and optimism as the first-order latent factors.

Prior studies both in organizational and educational contexts have indicated that PsyCap is positively correlated with adaptive outcomes and negatively correlated with maladaptive outcomes. For example, the predictive effects of PsyCap on adaptive outcomes, such as subjective wellbeing (Li et al., 2014; Datu and Valdez, 2016), academic engagement (King et al., 2020), intrinsic motivation (Siu et al., 2014), academic satisfaction (Ortega-Maldonado and Salanova, 2018), and academic performance (Datu et al., 2018; Carmona-Halty et al., 2019a,b; Martínez et al., 2019) were confirmed. On the other hand, the negative effects of PsyCap on maladaptive outcomes, such as academic procrastination (Hicks and Wu, 2015), substance abuse problems (Krasikova et al., 2015), and depressive symptoms (King and Caleon, 2021) were also confirmed. The control-value theory, as the framework for studying achievement emotions, holds that control and value appraisals are the two most important antecedents of individuals’ achievement emotions (Pekrun et al., 2002; Pekrun, 2006, 2009). Besides, considering that PsyCap is more stable than emotions (Luthans et al., 2007b) and individuals with higher PsyCap are more likely to experience positive emotions (Avey et al., 2011), it can be posited that PsyCap would be the predictor of achievement emotions in school contexts. Correspondingly, we formed the second hypothesis of this study.

*H2*: School PsyCap would be positively correlated with positive achievement emotions, such as enjoyment, and negatively related to negative achievement emotions, such as anxiety and boredom.

Besides, the job demand-resources theory holds that individual resources can not only positively affect the level of job engagement (Akanni et al., 2019), but also change the job demands into challenges (Siu et al., 2014). Since PsyCap is a kind of individual resource (e.g., economic capital, social capital, and human capital), we form the third hypothesis of this study.

*H3*: School PsyCap would have a positive predictive effect on academic engagement among Chinese college students.

## Materials and Methods

### Participants and Procedure

A total of 1,000 sophomores who learn English as a foreign language at a university in southwest China participated in this study. The mean age of the participants was 19.43 years (*SD* = 0.83 years). The sample comprised 215 males (21.5%) and 785 females (78.5%). The gender distribution in the sample is consistent with the gender ratio of normal universities in Mainland China.

The scales used in this study were firstly translated from English into Chinese, and then back-translated by bilingual researchers to ensure the equivalence of the Chinese version of these scales. The questionnaire consists of the school PsyCap scale, engagement and disaffection scale, and achievement emotion scale, which took about 25 min to complete. Participants completed the questionnaire in an English class under the guidance of their English teachers.

### Measures

#### School Psychological Capital

The Chinese version of the school PsyCap scale was adapted from King and Caleon’s (2021) school psychological capital scale. The school psychological capital scale is in English, and it was adapted from the psychological capital scale that was originally used to measure employees’ PsyCap capacity in industrial-organizational contexts (Luthans et al., 2007a). The important contribution of King and Caleon’s (2021) study was that the items in the psychological capital scale were replaced and adapted for the first time to be relevant to the school context. In their study (King and Caleon, 2021), five experts in psychology and education were asked to evaluate and classify the 24 items on the PsyCap scale and those items that received 80% of the experts’ approval would be retained. Finally, 16 items were retained to measure students’ school PsyCap capacity. The lowest acceptable factor loading is 0.40 (Matsunaga, 2010), and the factor loading of one item of the optimism scale in the present study (i.e., “Overall, I expect more good things than bad things to happen to me in school”) was below this criteria. Therefore, we adapted and translated King and Caleon’s (2021) 16-item school psychological capital scale into a 15-item Chinese school PsyCap scale.

This school PsyCap scale asks students to mark their agreement or disagreement with the descriptions, and the items were rated by a 7-point Likert scale (1 = strongly disagree to 7 = strongly agree). Sample items include as follows: “I feel confident that I can learn what is taught in school” (four items; self-efficacy); “if I have problems in school, I could think of many ways to solve them” (four items; hope); “I think I’m good at dealing with schoolwork pressures” (four items; resilience); and “I always look on the positive side of things in school” (three items; optimism).

#### Academic Engagement

Skinner and colleague’s engagement and disaffection scale (Skinner et al., 2009) were adapted to measure students’ behavioral and emotional engagement. The behavioral engagement subscale included four items, such as “I try hard to do well in English class,” and the emotional engagement subscale included four items, such as “English class is fun.” The cognitive engagement subscale was adapted from Reeve and Tseng’s (2011) Engagement Scale, an sample item is “when I study, I try to connect what I am learning with my own experiences.” The cognitive engagement subscale also consisted of four items. All three engagement subscales were rated on a 7-point Likert scale ranging from 1 (strongly disagree) to 7 (strongly agree).

In line with the previous studies (e.g., Lam et al., 2014), this study also treated engagement as a hierarchical construct, that is, academic engagement was posited as a second-order latent factor underpinned by the first-order latent factors of behavioral, emotional, and cognitive engagement. The Cronbach’s alpha reliability of the total engagement scale was 0.94. The Cronbach’s alpha reliability of each subscale of engagement was also examined, and the results showed that all the subscales had good reliability indices: behavioral engagement (*α* = 0.79), emotional engagement (*α* = 0.82), and cognitive engagement (*α* = 0.88). Moreover, the results of confirmatory factor analyses indicated the hierarchical model had excellent fit: (*χ*^2^ = 233.745; *df* = 51; *p* < 0.001; *χ*^2^/*df* = 4.58; RMSEA = 0.060 [90% CI: 0.052–0.068]; SRMR = 0.029; CFI = 0.971; and TLI = 0.963), which supported the hierarchical model of academic engagement.

#### Achievement Emotions

This study considered three concrete achievement emotions, namely, enjoyment, boredom, and anxiety because they are most frequently and intensely experienced by students during learning activities. According to Pekrun’s classification, enjoyment is perceived as positive emotions, while anxiety and boredom are classified as negative emotions (Pekrun, 2006; Pekrun and Linnenbrink-Garcia, 2012). We adapted items from Pekrun et al.’s (2011) achievement emotions questionnaire to measure students’ achievement emotions. Specifically, four items were used each to measure enjoyment, anxiety, and boredom. Answers were anchored on a 7-point Liker scale ranging from 1 (strongly disagree) to 7 (strongly agree). All subscales had good internal reliability: *α* = 0.837 for enjoyment, *α* = 0.835 for anxiety, and *α* = 0.763 for boredom.

### Statistical Analyses

We executed detailed item analyses to examine the psychometric properties of the school PsyCap scale. After this, Confirmatory Factor Analysis (CFA) models were conducted to examine the structure of the school PsyCap. We tested three models, namely, the unidimensional model, four-factor model, and hierarchical model, to determine which model fits the data well. A series of indices were used to evaluate model fit: root mean square error of approximation (RMSEA), comparative fit index (CFI), Tucker-Lewis index (TLI), and standardized root mean square error of approximation (SRMR). The values of CFI and TLI more than 0.90 mean that the model fit is adequate, and the values of RMSEA and SRMR less than 0.08 are regarded as acceptable (Hu and Bentler, 1999; Chen, 2007). Afterward, multigroup second-order CFA model was used to evaluate the measurement invariance of school PsyCap scale across gender groups.

To assess between-network validity, the zero-order correlations between school PsyCap and a wide range of academic outcomes, such as academic engagement, enjoyment, anxiety, and boredom, were tested. Subsequently, structural equation model (SEM) was conducted with PsyCap as predictor and engagement, enjoyment, anxiety, and boredom as outcome variables. Gender and age were considered as covariates.

## Results

### Treatment of Missing Data

This study used questionnaires of 1,000 Chinese sophomores as the analytical sample, and 0.4% of the questionnaires had missing responses. The expectation-maximization technique was applied to supplement the loss of information caused by missing responses.

### Psychometric Properties of the School PsyCap Scale

#### Item-Level Analyses

A set of item analyses were firstly conducted to assess the item quality of the school PsyCap scale. Precisely, the means and variance, the distributional properties, the corrected item-total correlation, and Cronbach’s alpha if item deleted of all the 15 items were analyzed and evaluated. The results of item-level analyses for the school PsyCap scale are shown in Table 1.

**Table 1:** Item-level analyses for the school PsyCap scale.

Item	Mean	*SD*	Skewness	Kurtosis	Corrected item-total correlation	Cronbach’s alpha if item deleted
EFF1	4.75	1.28	−0.51	−0.04	0.65	0.76
EFF2	4.58	1.31	−0.20	−0.24	0.61	0.75
EFF3	4.30	1.41	−0.16	−0.50	0.61	0.79
EFF4	4.61	1.19	−0.30	0.04	0.74	0.77
HO1	4.88	1.16	−0.31	−0.25	0.62	0.68
HO2	4.55	1.28	−0.37	−0.01	0.57	0.65
HO3	3.80	1.36	−0.04	−0.35	0.57	0.67
HO4	4.45	1.34	−0.18	−0.39	0.63	0.67
RES1	4.71	1.19	−0.25	−0.10	0.70	0.74
RES2	5.05	1.29	−0.51	−0.24	0.53	0.74
RES3	4.54	1.24	−0.24	−0.27	0.72	0.69
RES4	4.26	1.50	−0.05	−0.76	0.56	0.76
OPT1	4.93	1.27	−0.32	−0.35	0.50	0.64
OPT2	4.75	1.23	−0.32	−0.32	0.61	0.54
OPT3	4.30	1.43	−0.17	−0.44	0.62	0.57

EFF, self-efficacy; HO, hope; RES, resilience; and OPT, optimism.

Each item of the school PsyCap scale satisfies a normal distribution for both the skewness and kurtosis value conformed to the criteria suggested by Finney and DiStefano (2006), and the value of corrected item-total correlation of these items met the criteria proposed by Clark and Watson (1995). The skewness of all the items ranges from −0.04 to −0.51, which is within the criteria range of −2 to +2. The kurtosis values of all the items range from −0.76 to 0.04, which is also within the criteria range of −7 to +7. In addition, the values of corrected item-total correlations range from *r* = 0.50 to *r* = 0.74, which satisfy the criteria of *r >* 0.40.

The reliability (Cronbach’s alpha) of the total school PsyCap scale is *α* = 0.91. In the present study, the Cronbach’s alpha for each of the subscales of school PsyCap measure was 0.81 (self-efficacy), 0.73 (hope), 0.78 (resilience), and 0.68 (optimism). The results indicated that the total school PsyCap scale had good reliability and the reliability of the four subscales was also acceptable.

Subsequently, the four subscales’ Cronbach’s alpha reliability were checked again *via* item deletion. That is, we inspected whether the subscale’s Cronbach’s alpha reliability would increase or decrease by deleting one of its items. For instance, we looked at whether deleting the item “if I have problems in school, I could think of many ways to solve them” will increase or decrease the Cronbach’s alpha for hope. As shown in Table 1, we found that the 15 items of the school PsyCap scale would only result in lower Cronbach’s alpha reliability when any item deletion was conducted.

#### Confirmatory Factor Analysis

To further examine the psychometric properties of the school PsyCap scale, a series of confirmatory factor analyses were conducted. Specifically, three competing models were used to fit the data. Model 1 was a unidimensional model which posited that all the 15 items loaded into an omnibus school PsyCap construct. Model 2 was a four-factor model that had four inter-correlated latent constructs: self-efficacy, hope, resilience, and optimism. Model 3 was a hierarchical model that posited PsyCap as a second-order latent factor underpinned by the four first-order latent factors of self-efficacy, hope, resilience, and optimism.

As shown in Table 2, the model fit of the unidimensional model (model 1) was grudgingly acceptable. Both the four-factor model (model 2) and the hierarchical model (model 3) had an adequate fit. The target coefficient, which refers to the ratio of the chi-square of the first-order model to the chi-square of the higher-order model, is used to prove the existence of a higher-order construct and the upper limit of the target coefficient is 1 (Marsh and Hocevar, 1985). The target coefficient of the school PsyCap scale in this study is 0.90, indicating that this scale is a higher-order construct. Coupled with theoretical reasons (King and Caleon, 2021), the hierarchical model was adopted.

**Table 2:** CFA results of the three competing models.

Model	*χ*^2^	*df*	*χ*^2^/*df*	RMSEA	90% CI	CFI	TLI	SRMR
Model 1	587.108^***^	90	6.52	0.074	0.069, 0.080	0.920	0.907	0.040
Model 2	430.270^***^	84	5.12	0.064	0.058, 0.070	0.944	0.930	0.035
Model 3	476.006^***^	86	5.53	0.067	0.062, 0.073	0.937	0.923	0.037

*** *p* < 0.001.

### Measurement Invariance of School PsyCap Across Gender Groups

To examine the invariance of school PsyCap scale across gender groups, multigroup second-order CFA model was conducted. Followed by the principles of testing invariance in a second-order CFA model recommend by Wang and colleagues (Wang and Wang, 2019), this study firstly tested second-order configural invariance of the school PsyCap scale, and then tested the invariance of second-order factor loadings. Measurement invariance will be established if the following two requirements are satisfied: (1) the overall model fit is adequate (Little, 1997) and (2) the value of ΔCFI between two nested models should be smaller than or equal to 0.01 (Cheung and Rensvold, 2002).

#### Second-Order Configural CFA Model

In the present study, school PsyCap was viewed as a second-order construct with self-efficacy, hope, resilience, and optimism as the first-order latent factors. In the second-order configural CFA model, the measurement parameters and structural parameters can be freely estimated, and covariances between the residual terms of the first-order factors were all set to zero. Besides, for the purpose of model identification, intercepts of the first-order factors and the means of the second-order factors were all also set to zero.

The results showed that the second-order configural model fits the data well: *χ*^2^ = 611.640; *df* = 171; *p* < 0.001; *χ*^2^/*df* = 3.58; RMSEA = 0.072 [90% CI: 0.066–0.078]; SRMR = 0.041; CFI = 0.929; and TLI = 0.913. This indicated that the results of the configural model can be used as the baseline values against which the specified restricted models can be compared.

#### Testing Invariance of Second-Order Factor Loadings

The invariance of first-order factor loadings and item intercepts are the prerequisites for verifying the measurement invariance of the second-order factor loadings. Thus, both the invariance of the first-order factor loadings and item intercepts across gender groups would be firstly identified.

As shown in Table 3, the overall model fit was good and all ΔCFIs were smaller than 0.01, indicating that configural, metric, and scalar invariances were established in first-order factors of the school PsyCap.

**Table 3:** Testing first-order factor loadings and item intercepts across genders.

Model	*χ*^2^	*df*	CFI	ΔCFI	TLI	RMSEA	90% CI	SRMR
M1: Configural invariance	556.923	167	0.937	–	0.921	0.069	0.063, 0.075	0.039
M2: Metric invariance	582.338	178	0.935	0.002	0.923	0.068	0.062, 0.074	0.048
M3: Scalar invariance	633.885	193	0.929	0.006	0.923	0.068	0.062, 0.074	0.056

After checking the invariance of first-order factor loadings and the item intercepts, we tested the invariance of the second-order factor loadings by way of testing whether the relations between the four first-order factors (i.e., self-efficacy, hope, resilience, and optimism) and school PsyCap are invariant across gender groups. Specifically, we imposed equality restrictions on both first-order factors and second-order factors, and then compared model fit between the current model and the second-order configural CFA model. The model fit of current model was good: *χ*^2^ = 689.938; *df* = 200; *p* <0.001; *χ*^2^/*df* = 3.45; RMSEA = 0.070 [90% CI: 0.065–0.076]; SRMR = 0.060; CFI = 0.921; and TLI = 0.917. Comparing with the second-order configural CFA model, we got the following results: ΔCFI = 0.929–0.921 = 0.008 < 0.01. That is, the second-order factor loadings of school PsyCap were invariant across gender groups.

### School PsyCap as a Predictor of Academic Outcomes

The psychometric properties of the school PsyCap scale were sufficient for use among Chinese college students and confirmatory factor analysis indicated that the school PsyCap is best treated as a second-order latent variable. After this, we aimed to examine whether school PsyCap is positively linked to some key forms of academic outcomes.

#### Bivariate Correlations

The zero-order correlations between school PsyCap and several academic outcomes were examined. More specifically, we focused on the correlations between school PsyCap and academic engagement and three discrete achievement emotions (enjoyment, anxiety, and boredom). As expected, school PsyCap was positively related to academic engagement and positive emotions (i.e., enjoyment) and negatively correlated with negative emotions (i.e., anxiety and boredom). The results are presented in Table 4.

**Table 4:** Results of descriptive, bivariate correlations, and Cronbach’s alpha reliability.

S. No.		1	2	3	4	5	6	7
1.	School PsyCap	–	0.482^**^	0.401^**^	−0.236^**^	−0.200^**^	−0.079^*^	0.038
2.	Engagement		–	0.826^**^	−0.444^**^	−0.561^**^	0.130^**^	0.012
3.	Enjoyment			–	−0.511^**^	−0.596^**^	0.156^**^	−0.007
4.	Anxiety				–	0.747^**^	−0.110^**^	0.006
5.	Boredom					–	−0.202^**^	−0.003
6.	Gender						–	−0.139^**^
7.	Age							–
	Mean	4.57	4.51	4.44	3.61	3.34	1.79	19.41
	*SD*	0.87	0.91	1.07	1.26	1.22	0.41	1.01
	Cronbach’s alpha	0.91	0.94	0.84	0.84	0.76	–	–

* *p* < 0.05;

** *p* < 0.01.

#### Structural Equation Modeling

**Figure 1:**
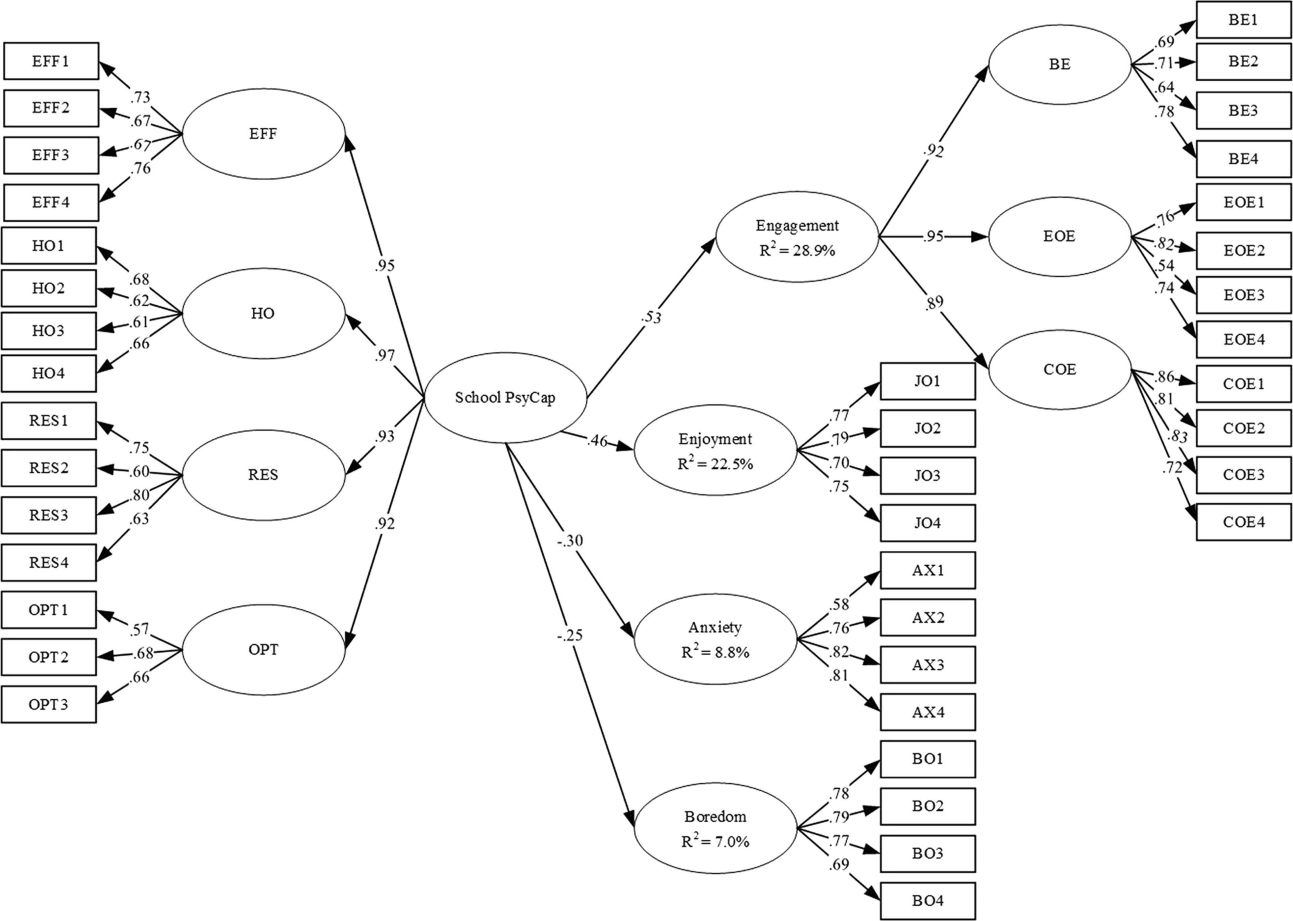
Final structural equation model on school PsyCap and academic outcomes. EFF, self-efficacy; HO, hope; RES, resilience; OPT, optimism; BE, behavioral engagement; EOE, emotional engagement; COE, cognitive engagement. All parameters in the figure are standardized coefficient and all paths are significant at *p* < 0.001. For brevity reason, the residuals are not presented in the figure. Gender and age are the covariates.

We constructed a SEM in which school PsyCap is an exogenous variable and academic engagement, enjoyment, anxiety, and boredom are outcome variables (see Figure 1). The model fit was good: *χ*^2^ = 2299.760; *df* = 755; *p* < 0.001; *χ*^2^/*df* = 3.05; RMSEA = 0.050 [90% CI: 0.047–0.052]; SRMR = 0.047; CFI = 0.912; and TLI = 0.905, which indicates that the proposed model fits the sample well. Results are showed in Figure 1. In line with our hypotheses, school PsyCap has positive predictive effect on academic engagement (*β* = 0.53, *p* < 0.001) and positive emotion (i.e., enjoyment; *β* = 0.46, *p* < 0.001). However, the predictive effect of school PsyCap on negative emotions is negative, particularly, anxiety (*β* = −0.30, *p* < 0.001) and boredom (*β* = −0.25, *p* < 0.001).

## Discussion

In the present study, we adapted and translated the existing school PsyCap scale (King and Caleon, 2021) into Chinese. Then, the psychometric properties of the 15-item school PsyCap scale were evaluated with Chinese college students as participants. Next, the association between school PsyCap and adaptive and maladaptive learning-related outcomes were examined. We found that the 15-item school PsyCap scale had good psychometric properties and the measurement invariance of this scale was also confirmed in the Chinese university context and that school PsyCap has a significant predictive effect on academic engagement and achievement emotions. This study contributes to the literature on PsyCap which has mainly been confined to the industrial-organization or western cultural contexts but has not yet appeared in the Chinese context.

By comparing three competing models of the school PsyCap scale, school PsyCap was suggested to be regarded as a higher-order latent variable underpinned by the four first-order latent variables of self-efficacy, hope, resilience, and optimism. Hypothesis one was supported. This result is consistent with prior studies on PsyCap in industrial-organization contexts (Larson and Luthans, 2006), Philippine context (Datu et al., 2018), and Singapore context (King and Caleon, 2021). In addition to the theoretical reasons proposed by King and Caleon (2021), the present study also evaluated the target coefficient of the school PsyCap scale to support the hierarchical conceptualization of school PsyCap. The four components of school PsyCap have extensively been explored in the educational context, and the significance of the present study lies in the validation that school PsyCap is a second-order latent variable with its four components as first-order latent variables.

The present study also mined both the positive and negative associations between school PsyCap and achievement emotions. Precisely, we found that school PsyCap has a positive predictive effect on positive achievement emotions, such as enjoyment. School PsyCap also negatively predicted negative achievement emotions, such as boredom and anxiety. The second hypothesis of this study was also confirmed. These findings were consistent with the previous studies that school PsyCap was positively correlated with adaptive outcomes, such as subjective wellbeing (Li et al., 2014), interdependent happiness, flourishing (Datu and Valdez, 2016), positive affect, and life satisfaction (King and Caleon, 2021). On the other hand, our findings were also consistent with prior studies that PsyCap negatively predicted maladaptive outcomes, such as emotional exhaustion, depersonalization (Ding et al., 2015), procrastination (Hicks and Wu, 2015), and depressive symptoms (King and Caleon, 2021). To our knowledge, this study is the first to explore and verify the association between school PsyCap and achievement emotions among Chinese college students. Achievement emotions are crucial to learning (Zull, 2006; Tyng et al., 2017), and it is of great value to promote college students’ achievement emotions by way of intervening their school PsyCap.

We also found that school PsyCap is the positive predictor of academic engagement, which fully supports the third hypothesis. Although the referents of academic engagement are not identical, the consistent finding of this study and the previous studies is that school PsyCap has a positive predictive effect on academic engagement. Taking Pilipino high school students as participants, Datu and Valdez (2016) utilized behavioral and emotional engagement to represent academic engagement and confirmed that PsyCap had positive predictive effect on academic engagement. Our finding is also consistent with King and Caleon’s (2021) study that school PsyCap positively predicated academic engagement. In King and Caleon’s (2021) study, the scores of behavioral, emotional, and cognitive engagement were aggregated to form an overall score of academic emotions. Academic engagement is one of the optimal academic-related outcomes, and exploring the link between school PsyCap and academic engagement would have positive implications for the enhancement of academic outcomes.

Overall, the present study contributes to the literature on school PsyCap by (1) validating the school PsyCap scale among Chinese college students and demonstrating that school PsyCap is best viewed as a higher-order variable, (2) confirming both positive and negative predictive effects of school PsyCap on achievement emotions, and (3) verifying the positive association between school PsyCap and academic engagement. Since previous PsyCap-related studies were mostly carried out in western contexts (e.g., Carmona-Halty et al., 2019b,c; Martínez et al., 2019), this study took Chinese college students as participants to explore the effectiveness of school PsyCap scale as well as the predictive effects of school PsyCap on achievement emotions and academic engagement. The findings of our study indicated that school PsyCap may also be an important resource in some non-western cultural contexts, such as the Chinese university context.

## Limitation and Directions for Future Research

Although this study expanded the application scope of the school PsyCap scale and explored the correlations between school PsyCap and achievement emotions and academic engagement, four limitations need to be addressed. Firstly, data for all the measures were self-reported and the risk of common method bias cannot be completely avoided. In addition to the self-reported data, future studies are suggested to collect data from teachers and peers to reduce the common method bias. Secondly, samples from more colleges/universities are needed to make the research more representative. Chinese colleges and universities recruit students from all provinces in China, that is, every single university includes college students that come from all provinces of China. It is a typical situation that there are more female students than male students in normal universities in China and the correlations between appraisal antecedents and achievement emotions are equivalent across genders (Pekrun, 2018). Nevertheless, the female students took a high proportion in the present sample, which calls for future studies to select more male students to balance the gender distribution. Thirdly, the present study only took Chinese college students as the survey sample. Although both the psychometric properties and the target coefficient of the school PsyCap scale were evaluated with Chinese college students as participants, future studies should include Chinese elementary and secondary school students to further broaden the application scope of the school PsyCap scale. Lastly, this study was correlational and thus causal conclusions cannot be drawn. Given that the main purpose was to demonstrate the validity of the school PsyCap scale in the Chinese context, the correlational nature of this study was a necessity. However, the relations among school PsyCap, achievement emotions, and academic engagement might be dynamic reciprocal, and thus cross-lagged panel design is suggested for future studies.

## Educational Implications

The validation of the 15-item school PsyCap scale indicates that college administrators and teachers can utilize this scale to assess college students’ school PsyCap capacity in Chinese settings. Also, the significant correlations between school PsyCap and achievement emotions and academic engagement suggest that nurturing students’ school PsyCap capacity might be one appropriate way to enhance their academic and wellbeing outcomes. Furthermore, the psychological capital is more measurable and malleable than the traditional human and social capital (Luthans et al., 2004), which implicates that the enhancement of students’ PsyCap capacity could be one more effective way for the development of their academic outcomes.

Although the research on promoting students’ PsyCap capacity has not yet appeared extensively (Carmona-Halty et al., 2019b), existing studies have shown that it was possible to develop a student’s PsyCap capacity by promoting the four components of PsyCap (Luthans et al., 2008, 2010). Firstly, relating today’s learning to tomorrow’s life is one possible way for teachers to enhance their students’ PsyCap capacity because high-hope individuals would have clear goals, replenish their willpower, and feel excited about their future (Marques et al., 2017). Secondly, teachers and educators are suggested to provide clear expectations for students (Eley and Stallman, 2014) as well as increase students’ control and choice over their studies (Brewer et al., 2019) to enhance students’ ability to bounce back when facing challenges or adversities. Thirdly, instructors can change the self-evaluation of those stressful students by, for example, listing solutions to their perceived stress to maintain an optimistic outlook about their studies (Rand et al., 2020). Fourthly, self-efficacy was believed to be the dominating component of human agency and teachers are the credible resources to nourish students’ self-efficacy beliefs by way of persuading and providing positive feedback so that students can experience mastery in learning (Usher and Pajares, 2008; Honicke and Broadbent, 2016).

The affective issues of learning activities are practically relevant to effective teaching (Kyriacou, 2009). Empirical studies show that students’ academic success is positively correlated with their positive achievement emotions and negatively correlated with the negative achievement emotions (e.g., Putwain et al., 2013; Dewaele et al., 2018), which indicates that increasing students’ positive achievement emotions (e.g., enjoyment) and reducing their negative achievement emotions (e.g., anxiety and boredom) would be the effective methods for teachers to motivate students to sustain further efforts in learning activities. The present study confirmed the correlation between school PsyCap and achievement emotions among Chinese college students; therefore, teachers and educators are suggested to influence on students’ achievement emotions by way of improving their psychological capital. In addition, the correlation between school PsyCap and academic engagement was also verified, which indicates that the enhancement of students’ psychological capital would contribute to effective teaching because students’ involvement and engrossment in learning activities would also be increased in this process (Fredricks et al., 2004).

## Conclusion

Given the significance of school PsyCap to education, the present study is the first to demonstrate the validity of the school PsyCap scale with Chinese college students as participants. Results also indicate that school PsyCap is a higher-order latent variable underpinned by first-order latent variables of self-efficacy, hope, optimism, and resilience. Besides, the predictive effects of school PsyCap on achievement emotions and academic engagement were also confirmed. Compared with other forms of capital (e.g., economic and social capital), it is more cost-effective to improve academic and wellbeing outcomes by developing the school PsyCap. Therefore, educators and teachers are suggested to nurture students’ school PsyCap capacity by creating environments and developing interventions.

## Data Availability Statement

The original contributions presented in the study are included in the article/supplementary material, further inquiries can be directed to the corresponding author.

## Ethics Statement

The studies involving human participants were reviewed and approved by the Human Research Ethics Committee of the University of Hong Kong. The patients/participants provided their written informed consent to participate in this study.

## Author Contributions

XK designed, analyzed the survey, and wrote the manuscript. YW conducted the survey and edited the manuscript. LL proofread the manuscript. All authors contributed to the article and approved the submitted version.

### Conflict of Interest

The authors declare that the research was conducted in the absence of any commercial or financial relationships that could be construed as a potential conflict of interest.
